# Performance Evaluation of Low-Cost Seismic Sensors for Dense Earthquake Early Warning: 2018–2019 Field Testing in Southwest China

**DOI:** 10.3390/s19091999

**Published:** 2019-04-29

**Authors:** Jihua Fu, Zhitao Li, Hao Meng, Jianjun Wang, Xinjian Shan

**Affiliations:** 1 Institute of Crustal Dynamics, China Earthquake Administration; Beijing 100085, China; zhitao.lee@163.com (Z.L.); wjj2855@vip.sina.com (J.W.); 2 School of Instrumentation Science and Optoelectronics Engineering, Beijing Information Science & Technology University, Beijing 100192, China; menghao@bistu.edu.cn; 3 State Key Laboratory of Earthquake Dynamics, Institute of Geology, China Earthquake Administration; Beijing 100029, China; xjshan@163.com

**Keywords:** earthquake, early warning, low-cost seismic sensor, field testing, MEMS

## Abstract

Earthquake Early Warning (EEW) was proved to be a potential means of disaster reduction. Unfortunately, the performance of the EEW system is largely determined by the density of EEW network. How to reduce the cost of sensors has become an urgent problem for building a dense EEW. A low-cost seismic sensor integrated with a Class C MEMS accelerometer was proposed in this paper. Based on minimal structure design, the sensor’s reliability was enhanced, while the costs were cut down as well. To fully reveal the performance, ten of the seismic sensors were installed and tested in Sichuan Province, southwest of China from May 2018 to February 2019. The seismic records obtained by the MNSMSs were compared with those by the traditional strong motion seismographs. The records obtained by the MNSMSs have good consistency with the data obtained by the Etnas. The MNSMSs can obtain clear seismic phases that are enough to trigger earthquake detections for EEW. By noise analysis, different channels of the same sensor and different sensors have good consistency. The tested dynamic range (over 87 dB) and useful resolution (over 14.5 bits) are completely in conformity with the designed parameters. Through real field testing, small earthquakes (M 3.1–3.6) can be detected by all three components E-W, N-S, and U-D within 50 km. In all, the low-cost seismic sensor proposed as a high-performance Class C MEMS sensor can meet the needs of dense EEW in terms of noise, dynamic range, useful resolution, reliability, and detecting capabilities.

## 1. Introduction

Of all natural disasters, the earthquake is one of the greatest threats to modern society. The Earthquake Early Warning (EEW) was thought to be a potential means of disaster reduction [1,2]. In recent years, a large number of research efforts have been devoted to the study of the EEW around the world. The EEW system of Mexico provided 60 s or more of warning time to Mexico City from an earthquake occurring 320 km away near the Guerrero coast in 1995 [3]. The Urgent Earthquake Detection and Alarm System (UrEDAS), a typical onsite EEW system in Japan, can issue an alarm a few seconds to 40 s before the arrival of destructive seismic waves [4,5]. EEW systems of Taiwan have been in operation since 1993, and are capable of sending predicted intensity and warning times to school attendees [6,7,8,9]. Besides, some other EEW systems were also developed and tested in many countries and regions, such as California [10,11], southern Italy [12], Southwest Iberia [13], India [14], and China [15,16].

The working principle of an EEW system is usually based on the idea that P-waves are faster than destructive S-waves, and the transmission speed of ground motion data are much faster than seismic waves. Therefore, EEW is a race against time. A closer-to-earthquake seismic sensor means that the estimated earthquake location and magnitude can be obtained soon after the arrival of P-waves, and the EEW system can serve more distant warning target areas. Therefore, the performance of an EEW system is greatly determined by the density of the EEW network. However, it will significantly increase the expense of EEW system to build a dense seismic monitoring network with the traditional strong motion seismograph or seismograph. To reduce instrument costs and establish high density monitoring networks, a new type of low-cost accelerometer based on the Micro-Electro Mechanical System (MEMS) was introduced to seismic applications since the 1990s [17].

Accelerometers are defined as Class A (useful resolution from about 20 to 26 bits with a dynamic range over 111 dB), Class B (useful resolution of about 16–19 bits over peak-to-peak ±2 g range), and Class C (useful resolution of about 12–15 bits over peak-to-peak ±2 g range) by the Advanced National Seismic System (ANSS) [18]. Although some other approaches were applied, such as Molecular-Electronic Transducers (MET), the class C MEMS sensors are a commercial low-cost solution for EEW indeed [19,20]. To understand their capabilities and limitations, some Class C MEMS sensors were tested for EEW applications [21,22,23]. And currently, several MEMS-based EEW systems were developed, including the Self-Organising Seismic Early Warning Information Network (SOSEWIN) [24], the Quake-Catcher Network (QCN) [25], the P-Alert network [26], and the EDAS-MAS [27], where QCN and P-Alert network use Class C MEMS sensors to collect seismic data. The data collected by QCN and P-Alert network provided valuable observations for EEW, intensity rapid intensity reporting (e.g. Shake Map), and some other research purposes. Though the 14-bit QCN sensors are sufficient for the previous example of finding the back-azimuth of a M4.7 earthquake at 11 km, the sensors are less capable of resolving a smaller or more distant earthquake [28]. The minimum number of stations needed to trigger an earthquake is set at 12 in the P-Alert network, which is much greater than that of the Central Weather Bureau (CWB) system, a traditional EEW system composed of force-balance accelerometers [29]. It might mean that the reliability of the outputs of the P-Alert sensors still need to be discussed, even for moderate earthquakes like M_L_ 6.4.

To resolve smaller and more distant earthquakes with reliable output, a low-cost seismic sensor integrated with a Class C MEMS accelerometer was proposed in this paper. The price of mass production is hopefully controlled below $300. For field testing, ten of the seismic sensors were installed in Sichuan Province, southwest of China from May 2018 to February 2019. During the test period, significant and numerous earthquakes were recorded and uploaded to a server in real-time. According to the collected data, the performance of the low-cost seismic sensor was evaluated in terms of noise, dynamic range, useful resolution, reliability, and other detecting capabilities.

## 2. Instrument Descriptions

**Figure 1:**
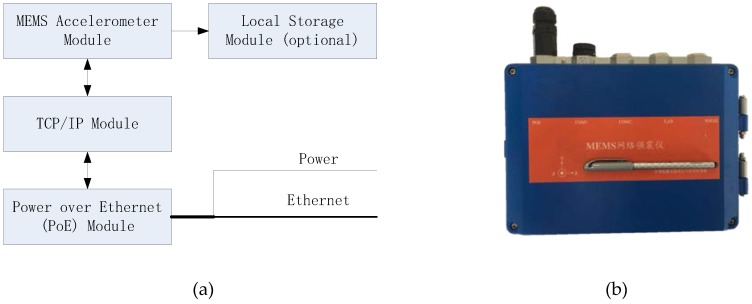
Composition and appearance of the low-cost seismic sensor: (**a**) Depiction of the sensor’s hardware modules; (**b**) Depiction of the sensor’s real appearance.

The low-cost seismic sensor proposed in this paper, so-called *MEMS Network Strong Motion Seismograph* (MNSMS), is equipped with a high-performance three-axis linear Class C MEMS accelerometer. To simplify hardware modules, enhance its reliability, and cut down on costs, the MNSMS is mainly composed of three or four hardware modules: the MEMS accelerometer module, TCP/IP module, Power over Ethernet (PoE) module, and local storage module (optional). The composition and appearance of the instrument are shown in Figure 1.

The MEMS accelerometer module is designed with a high-performance multiple serial ports Microcontroller Unit (MCU), a high-performance three-axis Class C MEMS accelerometer ADXL355, and a high-performance real-time clock (RTC). The MCU collects the data output of ADXL355 through a Serial Peripheral Interface (SPI). ADXL355 has an internal 20-bit, Σ-Δ analog-to-digital converter (ADC) and a built-in band-pass filter to fulfill the analog-to-digital conversion of an acceleration signal and the signal conditioning, where the pass-band of the built-in filter is set from 0.005 to 25 Hz. A simplified Network Time Protocol (NTP) was used to synchronize the RTCs of all MNSMS to the NTP server’s clock, and time stamps in the waveform data were generated with the help of the RTC and internal timer of MCU.

The TCP/IP module is a standalone communication unit of the MNSMS, which is applied to transmit ground motion data to a server by TCP/IP in real-time. The TCP/IP module is connected to the MEMS accelerometer module through a high-speed serial port, and accesses the Internet or Intranet via an Ethernet interface. The TCP/IP module can build and maintain a connection with a server automatically. Due to the introduction of the standalone TCP/IP module, the MEMS accelerometer module is liberated from the routines of the connection maintenance with the server, which makes it possible that an MCU-based system can control the whole low-cost seismic sensor rather than a complex ARM-based system. The MCU-based system is also cheaper and has lower power consumption than an ARM-based one. The PoE module is introduced to simplify the interface of the MNSMS by integrating power over Ethernet wires. And the local storage module is an optional device for the MNSMS, in which a circular queue is built for temporary storage of local continuous ground motion data. The ground motion data are written into the local storage module via another high-speed serial port of the MEMS accelerometer module. Some specifications of the MNSMS are listed in Table 1.

## 3. Performance Evaluation

### 3.1. Field Test Stations

**Figure 2:**
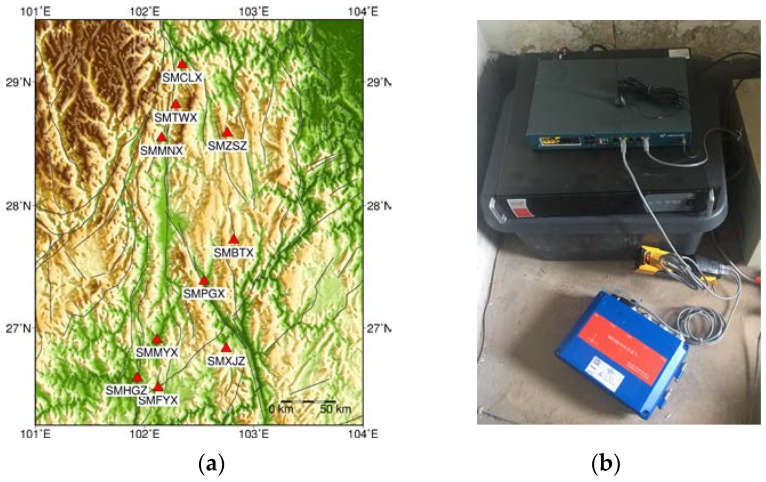
Locations and the station equipment of the tested MNSMSs: (**a**) Depiction of the MNSMSs’ locations; (**b**) Depiction of the station equipment serving for the MNSMSs.

Since May 2018, ten MNSMSs have been installed for field testing along the Anninghe fault and the Daliang Mountain fault in Sichuan province, southwest of China. The detailed information of the ten stations is listed in Table 2. The stations’ map and equipment of station are shown in Figure 2.

These MNSMSs were installed indoors on the ground. Their *x*-axis points east, *y*-axis points north, and the *z*-axis points upward. The station equipment included a 3G/4G router and an Uninterrupted Power Supply (UPS) at least to maintain the normal operation of the MNSMSs. By settings of 3G/4G routers, all MNSMSs were defined as a Virtual Private Network (VPN), each sensor was given a virtual fixed Internet Protocol (IP) address, and all sensors were maintained and managed through the IP addresses. With the VPN, a low-cost seismic sensor array was built based on mobile networks, and the ground motion data of the network were sent to a server located at Institute of Geology, China Earthquake Administration in real-time.

### 3.2. Noise Analysis

**Figure 3:**
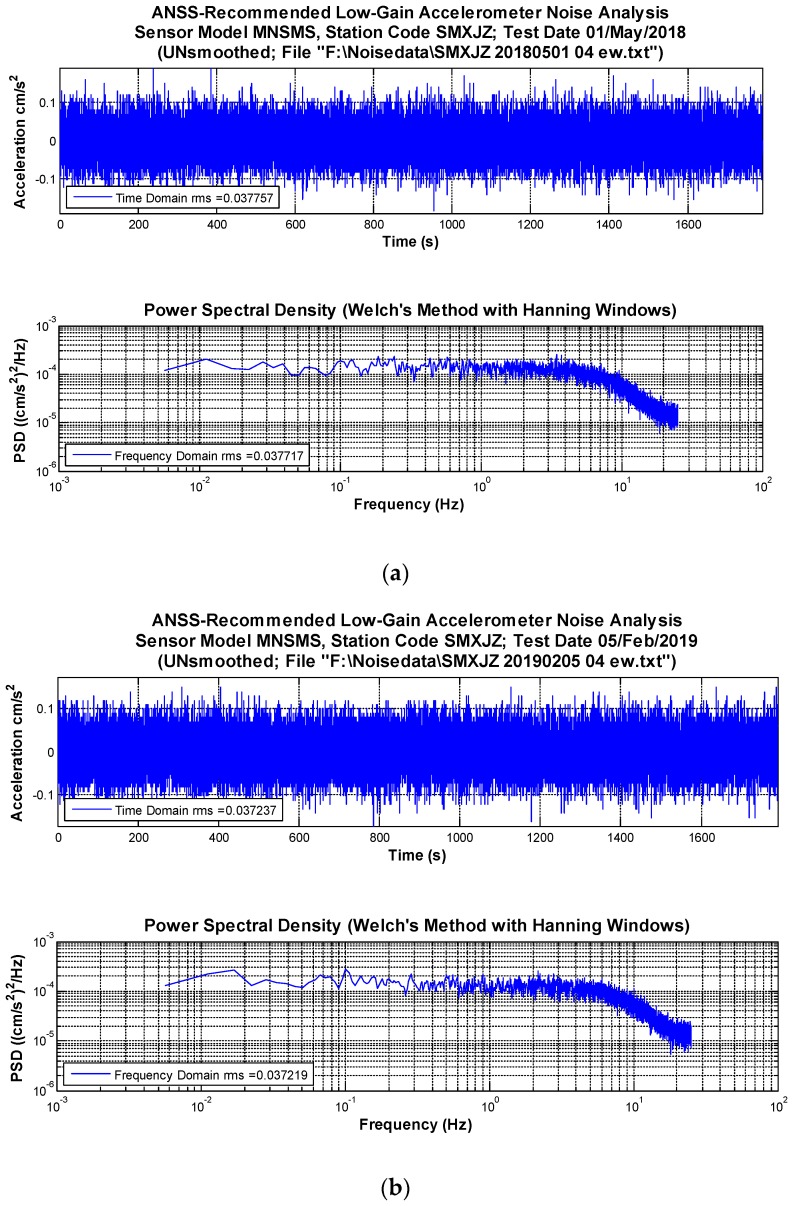
Noise signal of the sensor SMXJZ in time domain and their RMS, PSD calculated: (**a**) Analysis results of the noise signal obtained on May 1, 2018; (**b**) Analysis results of the noise signal obtained on February 5, 2019.

Noise analysis is one of the powerful tools to evaluate the performance of a seismic instrument, which can be used to obtain the self-noise, dynamic range, and useful resolution. ANSS-recommended sensor self-noise root mean square (RMS) and power spectral density (PSD) were calculated in this paper [30,31]. Two time points (May 2018 and February 2019) were selected, and on those time points self-noise was tested for each sensor respectively. Therefore, the reliabilities of the tested MNSMSs were estimated by the changes of their self-noise. According to the suggestion of ANSS, 30 min and 90,000 points for each channel at 50 sps were included in self-noise calculation in order to resolve low frequencies and optimize ensemble averaging. Analysis results of the data collected on May 1, 2018 and on February 5, 2019 for the same sensor SMXJZ are shown in Figure 3.

The acceleration data shown in Figure 3 are the raw data obtained by the sensor SMXJZ on May 1, 2018 at 4 am directly, where there is no distinct seismic wave included. The offset of acceleration data is equal to 0, and the peak-to-peak value is almost less than 0.1 cm/s^2^. Therefore, it can be supposed that the ambient ground motion is much less than the sensor noise, and the collected acceleration data simply represent the self-noise of the sensor. Based on this assumption, RMS and PSD are calculated. The RMS obtained in time domain is almost the same with the one obtained in frequency domain. As shown in Figure 3, the PSD values were not being smoothed by any filter. The PSD values are flat, and the considered bandwidth is from 0.005 to 25 Hz, where 25 Hz is the Nyquist frequency. Besides, analysis results for all the other sensors are shown in Table 3 as well.

In Table 3, the self-noise RMSs were calculated for each sensor by three channels: east-west (E-W), north-south (N-S), and up-down (U-D). For each sensor, RMSs of E-W and N-S channels were almost the same, and RMS of U-D channel were a little greater than the other two channels. That is consistent with the structural characteristics of the Class C MEMS accelerometer. For the test performed around May 2018, RMSs of the same channels for different sensors were quite similar, except for the sensor SMMNX. The same type of Class C MEMS accelerometers were used in all the ten MNSMSs. The reason for this is that the effective digit of sensor SMMNX was set to only one digit after the decimal point, while the effective digits of others were set to two digits by contrast. Since the RMSs of the MNSMSs on each channel are always less than 0.1 cm/s^2^, the effective digits of the MNSMS should be set to two digits after the decimal point. So, the effective digit of sensor SMMNX should be the same with others’. Regardless of the sensor SMMNX, the RMSs of E-W and N-S channels were about 0.038 cm/s^2^, while the RMSs of UD channels were around 0.056 cm/s^2^. The maximum difference of RMSs between different sensors was 0.004 cm/s^2^, whose results showed that the nine sensors with the same effective digits had good coherence in terms of self-noise.

In Table 3 for the test performed around February 2019, RMSs of the same channels were also calculated for each sensor. The effective digits of sensor SMMNX was fixed. Similar results to May 2018’s were obtained: the RMSs of E-W and N-S channels were about 0.038 cm/s^2^, while that of UD channels was around 0.056 cm/s^2^. The maximum difference of RMSs between different time points was also 0.004 cm/s^2^. After nine months of continuous work, the self-noise of the MNSMSs did not change much, which showed that the performance of the MNSMS was stable and reliable.

When RMSs of the MNSMSs were obtained, their dynamic ranges were figured out via Equation (1)

(1) $$D=20\cdot \mathrm{lg}\frac{A_{\max}}{\mathrm{RMS}\times \sqrt{2}}$$

where *D* is the dynamic range. *A*_max_ is the measurement range of the MNSMSs and it also can be called clip level. RMS is the measured root mean square of the sensor’s self-noise.

When the dynamic range is given, the useful resolution can be obtained by Equation (2)

(2) $$N_{u}=\frac{D}{20\times \mathrm{lg}2}$$

where *N_u_* is the useful resolution of the MNSMS.

By the Equations (1) and (2), the dynamic range and the useful resolution of the MNSMSs were evaluated based on the ground motion data obtained around May 2018 and February 2019. As mentioned above, the measurement range of the MNSMSs is ±2 g. Since the sensors were installed in the southwest of China, the gravity acceleration g was set to 9.7913 m/s^2^ [32]. The analysis results are shown in Table 4.

As shown in Table 4, the dynamic range of the MNSMSs was between 90.57 and 91.46 dB in E-W and N-S directions. Correspondingly, the useful resolution of the MNSMSs varied in the range of 15.0 to 15.2. When it came to U-D direction, the dynamic range of the MNSMSs was between 87.56 and 88.18 dB, and the useful resolution varied from 14.5 to 14.6.

### 3.3. Earthquake Detections

**Figure 4:**
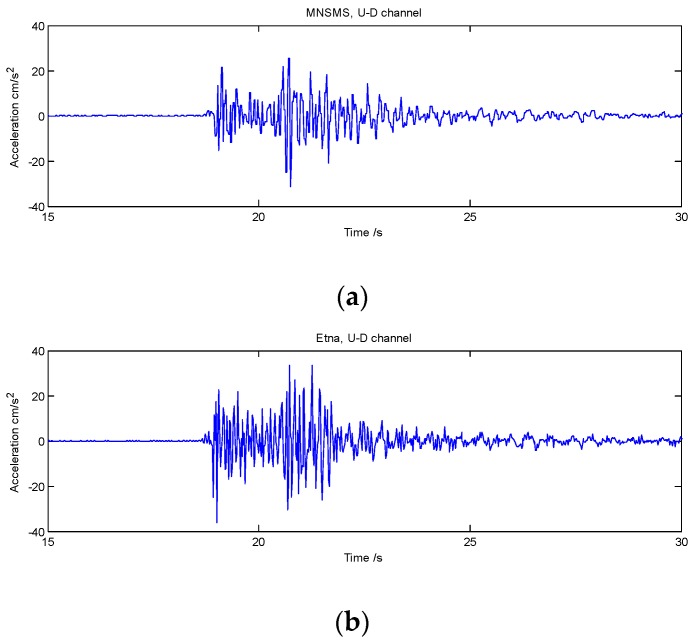
U-D channel records of earthquake M 4.3 occurred at Lat. 29.2° and Lon. 102.3° on May 16, 2018 4:46 pm obtained by the sensor SMCLX and the sensor Etna: (**a**) U-D channel record was obtained by the sensor SMCLX. (**b**) U-D channel record was obtained by the sensor Etna.

Significant and numerous earthquakes have been recorded since May 2018. Firstly, the seismic records obtained by MNSMSs and traditional strong motion seismographs were compared. The U-D channel records of earthquake M 4.3 occurred at Lat. 29.2° and Lon. 102.3° on May 16, 2018 4:46 p.m. obtained by the sensor SMCLX and a sensor Etna are shown in Figure 4, respectively. The sensor Etna is one kind of high precision, force-balanced strong motion seismograph, which was installed at Lat. 29.13° and longitude 102.34°, quite close to the sensor SMCLX.

As shown in Figure 4a,b, the U-D channel records obtained by the sensor SMCLX have good consistency with the data obtained by the sensor Etna. However, because the sampling rate of the sensor SMCLX is only 50 Hz and the sampling rate of the sensor Etna is 200 Hz, the sensor SMCLX lost some high frequency (>25 Hz) information comparing with the sensor Etna. Nevertheless, the sensor SMCLX obtained clear seismic phases, which was enough to trigger earthquake detection for EEW.

**Figure 5:**
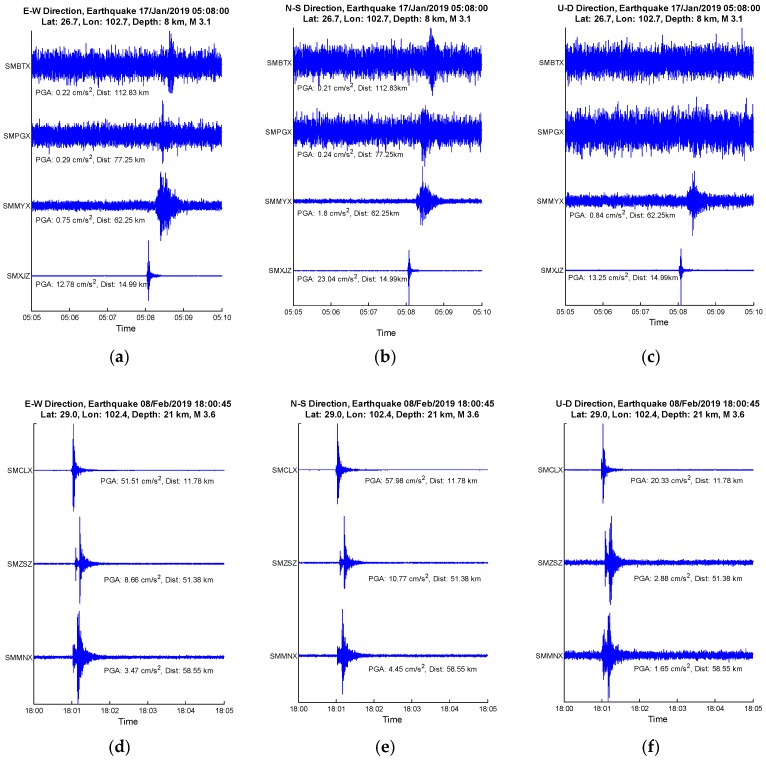
Ground motion records by the MNSMS network: (**a**–**c**) E-W, N-S, and U-D directions of ground motion records for the earthquake M 3.1 occurred at Lat. 26.7° and Lon. 102.7° on January 17, 2019 5:08 am respectively; (**d**–**f**) E-W, N-S, and U-D directions of ground motion records for the earthquake M 3.6 occurred at Lat. 29.0° and Lon. 102.4° on Feb. 8, 2019 6:00 pm respectively.

Secondly, two relatively small earthquakes were selected to reveal the seismic detection capabilities of the MNSMS, which are shown in Figure 5. In order to display small seismic waveforms, the Y-axis data were self-adapted to their PGAs, and the PGAs with unit were figured out.

As shown in Figure 5a–c, the earthquake M 3.1 can be detected by the sensor SMBTX about 112 km away. But for EEW application, P-waves should be detected by U-D component to estimate the magnitudes of destructive earthquakes [10]. In this way, both the sensor SMBTX and the sensor SMPGX about 77 km away failed to detect the earthquake M 3.1 by their U-D components, while the sensor SMMYX about 62 km away did detect the earthquake M 3.1 by its U-D component. Similarly, by Figure 1d–f, the earthquake M 3.6 was detected by the sensor SMMNX about 58 km away. To further reveal the detecting capabilities of the MNSMS, some other minor or teleseismic events detected are listed in Table 5.

The ground motion records shown in Table 5 are the detections by all E-W, N-S, and U-D components. And the sensors that detected the seismic events but failed to detect them by U-D components were also not listed in Table 5. The earthquake events 1 and 2 with the same magnitude M 3.2 can be easily identified by sensors within or around 20 km. And because of good self-noise performance, these two minor seismic events can also be detected by the MNSMSs over 50 km or even 70 km away. Furthermore, the earthquake event 3 and 4, two teleseismic events were detected by the tested MNSMS network in distances over 200 km or even 270 km. Due to the misconfiguration, the PGAs of station SMMNX have only one effective digit after decimal point for event 1, 2, and 3.

## 4. Conclusions

To build the dense EEW system, a low-cost seismic sensor integrated with a Class C MEMS accelerometer was proposed in this paper. On the basis of guaranteeing performance, the minimal structure idea was applied to the hardware and software design, which can enhance MNSMSs’ reliability and cut down costs as well. To fully reveal the performance, ten of the seismic sensors were installed and tested in Sichuan Province, southwest of China from May 2018 to February 2019.

The seismic records obtained by the MNSMSs were compared with those by the traditional strong motion seismographs were compared. The records obtained by the MNSMSs have good consistency with the data obtained by the Etnas. Although the sampling rate (50 Hz) of the MNSMSs is lower than the sampling rate (200 Hz) of the Etnas, the MNSMSs can obtain clear seismic phases which are enough to trigger earthquake detections for EEW.

Based on the collected data, the ANSS-recommended self-noise of the MNSMS was obtained. RMSs of E-W and N-S channels were about 0.038 cm/s^2^, while the RMSs of UD channels were around 0.056 cm/s^2^. And in the bandwidth considered from 0.005 to 25 Hz, the PSD are flat, which revealed that performances of the MNSMSs are homogeneous in frequency domain. Through noise analysis, for each sensor, RMSs of E-W and N-S channels were almost the same. It means that the two horizontal components E-W and N-S have good consistency with each other. As for different MNSMSs, their self-noise is comparatively consistent. This indicates that different sensors have good homogeneity. And after nine months of continuous work, the self-noise of the MNSMSs did not change much, which illustrates that the performance of the MNSMS is stable and reliable.

By the RMSs obtained in noise analysis and the known measurement range, the dynamic range of the MNSMSs was figured out, which was greater than 90 dB in E-W and N-S directions, and was greater than 87 dB in U-D direction. Correspondingly, the useful resolution of the MNSMSs was also obtained, which was greater than 15.0 bits in E-W and N-S directions, and was greater than 14.5 bits in U-D direction. The tested dynamic range and useful resolution are completely in conformity with the designed parameters of the MNSMS.

According to the seismic events detected by the MNSMS network from May 2018 to February 2019, even earthquakes with small magnitude M 3.1 to M 3.6 can be easily identified by the MNSMSs within or around 20 km. These small earthquakes can also be detected by the sensors proposed in this paper when the epicenter is within 50 km by all components E-W, N-S, and U-D, and over 50 km the seismic signal in U-D direction may be submerged by the self-noise of the MNSMS, which can cause failed detection. And for big teleseismic events with bigger magnitude than 4.7, can be detected by the sensors proposed through all three components over 200 km away.

In all, the real field test results show that the MNSMS proposed in this paper as a high-performance Class C MEMS sensor can meet the needs of dense EEW in terms of noise, dynamic range, useful resolution, reliability, and detecting capabilities. In view of its performance, the proposed sensor can also be used in some other seismic monitoring applications, such as rapid intensity reporting, automatic earthquake emergency handling, and structure health monitoring.

## Figures and Tables

**Table 1:** The specifications of the MNSMS.

Parameter	Value	Unit
Measuring Range	± 2	g (gravity acceleration)
Sampling Rate	50	Hz
Bandwidth	DC to 25	Hz
Dynamic Range	>84	dB
Useful Resolution	>14	bits
Nonlinear Relative Error	<1	%
Sensitivity Relative Error	<5	%
Power Consumption	<1.2	Watt

**Table 2:** Detailed station information of the low-cost seismic sensor network.

No.	Station Code	Latitude	Longitude	Field Type	Environment	Direction	Network
1	SMTWX	28.81	102.28	on the ground	Indoor	*x*-East *y*-North *z*-Up	3G/4G mobile networks (VPN)
2	SMFYX	26.50	102.12				
3	SMXJZ	26.83	102.74				
4	SMCLX	29.14	102.34				
5	SMMYX	26.89	102.11				
6	SMPGX	27.38	102.54				
7	SMBTX	27.71	102.81				
8	SMHGZ	26.54	101.93				
9	SMZSZ	28.59	102.50				
10	SMMNX	28.55	102.16				

**Table 3:** Self-noise RMS of the MNSMSs.

No.	Station Code	Testing Time: May 2018			Testing Time: February 2019		
		RMS/cm/s^2^			RMS/cm/s^2^		
		E-W	N-S	U-D	E-W	N-S	U-D
**1**	**SMBTX**	0.037	0.037	0.055	0.036	0.036	0.054
**2**	**SMCLX**	0.041	0.041	0.055	0.039	0.038	0.054
**3**	**SMFYX**	0.039	0.038	0.058	0.038	0.038	0.058
**4**	**SMHGZ**	0.038	0.039	0.057	0.038	0.037	0.056
**5**	**SMMNX**	0.013	0.013	0.031	0.037	0.037	0.054
**6**	**SMMYX**	0.040	0.039	0.057	0.039	0.038	0.056
**7**	**SMPGX**	0.038	0.038	0.056	0.038	0.038	0.056
**8**	**SMTWX**	0.038	0.037	0.054	0.037	0.037	0.054
**9**	**SMXJZ**	0.038	0.038	0.057	0.037	0.038	0.057
**10**	**SMZSZ**	0.037	0.037	0.056	0.041	0.040	0.058

**Table 4:** Estimated dynamic range and useful resolution of the MNSMSs.

Directions	Measured RMS/cm/s^2^	Dynamic Range/dB	Useful Resolution/bits
E-W	0.037–0.041	90.57–91.46	15.0–15.2
N-S	0.037–0.041	90.57–91.46	15.0–15.2
U-D	0.054–0.058	87.56–88.18	14.5–14.6

**Table 5:** Some minor or teleseismic events detected by the tested MNSMS network.

Event No.	Date and Time	Lat./°	Lon./°	Dep./km	Mag.	Station Code	Distance/km	PGA/cm/s^2^ E-W/N-S/U-D
1	02/May/2018 04:28:44	28.5	102.7	21	3.2	SMZSZ	21.95	2.76/2.66/1.25
						SMMNX	53.05	0.8/0.8/0.4
2	16/May/2018 16:44:02	29.2	102.3	9	3.2	SMCLX	7.72	45.64/49.47/12.14
						SMMNX	73.55	1.7/1.5/0.6
3	03/Jan/2019 08:48:06	28.2	104.9	15	5.3	SMBTX	212.37	0.99/0.97/0.4
						SMMNX	270.86	0.6/0.7/0.3
4	24/Feb/2019 05:38:10	29.5	104.5	5	4.7	SMTWX	228.81	0.65/0.63/0.48
